# Oxidative stress and decreased tissue HSP70 are involved in the genesis of sepsis: HSP70 as a therapeutic target

**DOI:** 10.5935/0103-507X.20200084

**Published:** 2020

**Authors:** Maicon Machado Sulzbacher, Mirna Stela Ludwig, Thiago Gomes Heck

**Affiliations:** 1 Research Group in Physiology, Department of Life Sciences, Universidade Regional do Noroeste do Estado do Rio Grande do Sul - Ijuí (RS), Brazil.; 2 Postgraduate Program in Comprehensive Health Care, Department of Life Sciences, Universidade Regional do Noroeste do Estado do Rio Grande do Sul - Ijuí (RS), Brazil.

**Keywords:** Sepsis, HSP70, Oxidative stress, Early diagnosis, Sepse, HSP70, Estresse oxidativo, Diagnóstico precoce

## Abstract

Sepsis is a systemic infection that causes multiple organ dysfunction. HSP70 is a protein responsive to cell stress, in particular oxidative stress. Therefore, this literature review sought to investigate the roles of HSP70 and oxidative stress in the pathophysiology of sepsis and the possibility of HSP70 as a therapeutic target. HSP70 exerts a protective effect when located in cells (iHSP70), and its decrease, as well as its increase in the extracellular environment (eHSP70), under oxidative stress is a biomarker of sepsis severity. In addition, therapies that increase iHSP70 and treatment with HSP70 promote sepsis improvement.

## Abstract (pt)

A sepse é uma infecção sistêmica que acarreta disfunção múltipla dos órgãos. A HSP70 é uma proteína responsiva ao estresse celular, assim como o estresse oxidativo. Esta revisão da literatura buscou investigar a HSP70 e o estresse oxidativo quanto à fisiopatologia da sepse e ao papel da HSP70 como possível alvo terapêutico. A HSP70 exerce efeito protetor quando localizada na célula (iHSP70), e sua diminuição, assim como seu aumento no ambiente extracelular (eHSP70) e o estresse oxidativo, é um biomarcador de gravidade na sepse. Além disso, terapias que aumentam a iHSP70 ou o próprio tratamento com HSP70 promovem a melhora na sepse.

## INTRODUCTION

Sepsis is a disseminated infection characterized by injury and systemic dysfunction^(1)^ and is considered a disease of great impact in the health arena. Annually, sepsis affects 30 million people, resulting in 6 million deaths.^(2)^ When analyzing the cost of hospitalization in a Brazilian hospital, the investment per patient was BRL 38,867.60, and 59% of this total amount was allocated to patients who died.^(3)^

The pathophysiology of sepsis is complex, and it is well established that the early diagnosis and treatment of sepsis is the main tool to obtain a higher survival rate. In view of this, the scientific community has sought to establish new forms of diagnosis and new therapeutic interventions.^(1,4)^ However, advances are needed to enable better patient prognosis.^(5)^

The investigation of the relationship between systemic infection, immune response and dysfunction in different organs is important for understanding the mechanisms by which the invading agent promotes generalized dysfunction in the body.^(6)^ Preventive interventions or treatment measures are often investigated in sepsis, both in clinical cases of patients and in experimental models.^(7,8)^ In this sense, the role of oxidative stress and the response to cell stress in sepsis have been studied, especially the expression of 70 kDa heat shock proteins (HSP70).^(9,10)^

This review aimed to verify the role of HSP70 and oxidative stress in the main dysfunctions that occur in sepsis, in addition to the possibility of HSP70 as a possible therapeutic target.

## METHODS

A narrative review was conducted by searching the Scientific Electronic Library Online (SciELO) and PubMed® databases with the terms “Sepsis”, “Sepsis and Heat Shock Protein 70” and “Sepsis and oxidative stress” from Medical Subject Headings (MeSH), from 2009 to September 2019, for studies in which the subjects sepsis and oxidative stress and sepsis and HSP70 were addressed.

### Sepsis: general aspects and its relationship with oxidative stress and HSP70

In sepsis, the imbalance among inflammation, coagulation, and fibrinolysis results in disseminated inflammation, microvascular thrombosis, endothelial injury, and systemic coagulopathy, which lead to decreased tissue perfusion and generalized dysfunction.^(11)^ To demonstrate this systemic dysfunction, a new form of diagnosis for sepsis was established in 2016, namely, a suspected or documented infection accompanied by an acute increase of 2 or more points in the Sequential Organ Failure Assessment (SOFA) score, considering dysfunctions in the respiratory, cardiovascular, coagulation, neurological, hepatic, and renal systems.^(1)^

The onset and worsening of sepsis and its worse prognosis are associated with increased oxidative stress^(1,12)^ in addition to alterations in HSP70, both in its expression (iHSP70) and in its extracellular levels (eHSP70).^(13)^

When there is an imbalance in the homeostasis of an organism leading to the onset of sepsis, there is an overproduction of reactive oxygen species (ROS). This increase in ROS requires an increase in the activity of the plasma and tissue antioxidant enzymes superoxide dismutase (SOD), catalase (CAT), and glutathione peroxidase (GPx) in an attempt to fight oxidative damage. However, in addition to being reduced throughout the pathology-induced condition, these antioxidant defenses cannot neutralize the excessive amount of ROS produced during sepsis. This imbalance establishes a condition of oxidative stress, which can cause oxidative tissue damage and is observed through cellular damage to lipid components (lipoperoxidation), proteins (protein carbonylation) or DNA,^(14)^ which have primordial roles in pathological processes, which, in turn, lead to dysfunction in the different organs affected in sepsis.^(14-16)^

Oxidative stress can cause mitochondrial damage, which leads to reduced tissue respiratory capacity due to partial decoupling of mitochondrial oxidative phosphorylation, leading to low levels of intracellular energy currency - adenosine triphosphate (ATP) - and increased blood lactate levels,^(17)^ which is considered a predictor of mortality in sepsis.^(18)^ This cellular energy insufficiency is related to the release of calcium ions from intracellular reserves and the onset of apoptosis, which contributes to the functional disorder of multiple organs in sepsis.^(19)^

Infection and oxidative stress can trigger cellular responses to stress in different cells and organs, altering the expression of specific proteins related to the protection of the organism. In this sense, proteomic studies are being used to understand different infectious conditions.^(20)^ The role of heat shock proteins (HSPs), especially the HSP70 family, has been studied in sepsis.^(21)^ The 72 and 73 kDa isoforms are highlighted and considered inducible (HSP72) and constitutive (HSP73 or Hsc70) isoforms.^(22)^

The heat shock response (HSR), capable of inducing the synthesis of HSP70, is one of the most conserved cytoprotection methods known and is utilized by simple organisms, such as bacteria, and by complex organisms, such as humans.^(22)^ HSP70 in the intracellular environment (iHSP70) has chaperone activity, correcting damaged proteins, and is able to assist in protein folding and refolding, modulating the inflammatory response, thus playing an important role in the response to cell stress.^(23)^

iHSP70 may play a protective role in an age-dependent response to sepsis, preventing apoptosis and intestinal, pulmonary and leukocyte inflammation.^(8)^ However, when this protein is observed in immune cells exposed to bacterial endotoxins (lipopolysaccharides - LPS), there is an increase in the export ratio of iHSP70 to the extracellular environment (eHSP70).^(24)^

During exposure to LPS, cells of the immune system increase the concentration of HSP70 in microvesicles responsible for carrying content to the cell membrane for future export (exosomes), and this concentration is even higher when the body temperature is high (39.5°C)^(24)^ as well as in the classic case of fever after bacterial infection. The levels of eHSP70 are proportionally related to plasma levels of LPS antigen.^(25)^

Serum eHSP70 and oxidative stress levels are high in patients with sepsis. When evaluated in the blood of patients with sepsis, these 2 variables can be considered biomarkers of sepsis severity and mortality.^(9)^ In individuals who die, eHSP70 increases with clinical worsening, and this increase is associated with increased plasma oxidative damage.^(9)^ By observing the correlation between eHSP70 and oxidative damage, eHSP70 itself can suffer oxidative damage (oxHSP70).^(10)^ An *in vitro* study showed that oxHSP70 is able to decrease the production of tumor necrosis factor alpha (TNF-α) by macrophages, a process that also decreases phagocytic activity and proliferation and impairs the immune response - precisely the opposite of what occurs when these cells are treated with eHSP70. Together, these data suggest that the concomitant cellular response to stress and systemic oxidative damage can accurately represent the severity of sepsis.^(10)^

### HSP70, oxidative stress and cardiovascular dysfunction in sepsis

Cardiovascular dysfunction in sepsis is the main cause of clinical worsening and consequent death.^(26)^ The exposure of cardiomyocytes to LPS promotes oxidative stress, which leads to greater caspase expression, with consequent cellular apoptosis and worsening of the inflammatory status.^(27)^

The proinflammatory cytokine interleukin 1 beta (IL-1β), which is synthesized in response to bacterial recognition by leukocytes, induces the synthesis of the enzyme inducible nitric oxide synthase (iNOS), which increases the production of nitric oxide (NO). Nitric oxide plays a key role in the establishment of hemodynamic changes, increasing vascular tone and permeability, and is associated with decreased blood pressure in sepsis.^(21)^ Furthermore, sepsis decreases the arterial contractile capacity in response to noradrenergic stimulation, causing impairment in vascular homeostasis related to hypotension and septic shock.^(28)^

Treatments for sepsis aim to restore tissue perfusion, with measures focusing on restoring and maintaining the hemodynamic status, oxygenation and organic function. The implications of intensified efforts in the search for successful innovative approaches for the treatment of myocardial dysfunction in sepsis may be considerable with regard to better patient care, resulting in lower mortality.^(26)^

Supplementation with the immunonutrient glutamine increases the levels of the HSP70 transcription factor (heat shock factor *-* HSF1), which increases the levels of HSP70 in cardiomyocytes, promotes a decrease in a marker of muscle injury (lactate dehydrogenase - LDH) and provides cardiac protection in sepsis.^(29)^ The increased expression of cardiac HSP70 suppresses the expression of proinflammatory and proapoptotic factors and nuclear factor kappa B (NF-κB) and iNOS activity,^(21)^ which increases cardiac function^(30)^ and mean arterial pressure (MAP) and contributes to vascular homeostasis in sepsis, which decreases mortality in an experimental model of the disease.^(30,31)^ In addition, it attenuates cardiac muscle injury, as observed by decreased levels of creatine phosphokinase (CPK), serum LDH and cardiac cell death.^(32)^

iHSP70 is also associated with increased levels of the antioxidant enzyme glutathione reductase (GSH) and decreased levels of oxidants (superoxide anion) in cardiac tissue. This protein promotes cardiovascular improvement and is associated with increased expression of the enzyme heme oxygenase (HO-1), which is involved with angiogenesis, and decreased expression of plasminogen activator inhibitor-1 (PAI-1), which attenuates the risk of intravascular thrombosis and, consequently, increases survival in an experimental model of sepsis.^(33)^

### HSP70, oxidative stress and neurological dysfunction in sepsis

The brain has always been considered a primordial organ due to the breadth of its functions, and changes in its functioning can trigger deleterious and fatal repercussions. As a result of the disease, patients with sepsis may present with neurological symptoms, ranging from delirium and confusion to coma.^(34)^

The interaction between the invading pathogen and the neurological system causes a cascade of events that leads to injury in the central nervous system, characterizing septic encephalopathy. These events include tissue hypoperfusion, mitochondrial dysfunction, energy imbalance, apoptosis and an amplified inflammatory response.^(35)^

The Glasgow Coma Scale (GCS), which verifies the level of consciousness, and electroencephalography (EEG), which checks brain electrical activity, have been tools used for the diagnosis of septic encephalopathy.^(36)^ Oxidative stress is highlighted in the pathophysiology of neurological dysfunction in sepsis, as it is associated with acute brain inflammation, cognitive deficit and long-term neurodegeneration in rats that survive sepsis.^(37)^ Furthermore, oxidative stress is associated with a bioenergetic imbalance, which leads to mitochondrial dysfunction and, in turn, to cell death and brain tissue damage in sepsis.^(38)^

In sepsis, there is increased expression of receptor for advanced glycation end products (RAGE), which is present in different cells and is related to proinflammatory signaling. This receptor has a soluble isoform (sRAGE), which can be found in the blood, and is correlated with the mortality of patients with sepsis.^(39)^ In brain tissue, RAGE may be present in the vascular endothelium, neurons and dendritic cells, as well as in monocytes that try to fight local infection. In sepsis, there is an increase in RAGE expression in the brain; this increase in RAGE expression is associated with the increased expression of the proinflammatory cytokines IL-1β, IL-6 and TNF-α, which cause brain inflammation; furthermore, it is associated with decreased expression of cerebral HSP70, which coincides with the accumulation of beta-amyloid peptide (Aβ) and increased tau protein phosphorylation, which, together with the aforementioned events, cause neurological dysfunction with cognitive worsening.^(39)^

As there is a decrease in the concentration of cerebral iHSP70 in sepsis, worsening of the disease can be observed.^(40)^ This decrease in iHSP70 in the brain is associated with increased NF-κB activity, resulting in inflammation and apoptosis in this tissue.^(41)^ However, when glutamine supplementation was performed preventively to induce sepsis in an experimental model, there was an increase in cerebral iHSP70 together with a decrease in NF-κB activity and apoptosis in the same tissue.^(41)^ With the same purpose, thermal therapy to prevent sepsis, which induces greater synthesis of cerebral iHSP70, affects the reduction in neurological dysfunction, which is measured by EEG, and attenuates the encephalopathy associated with sepsis in an experimental model.^(42)^

### HSP70, oxidative stress and respiratory dysfunction in sepsis

Lung injury occurs early in sepsis. The same occurs due to the inflammatory stimulus caused by invading microorganisms in the respiratory system, promoting vascular endothelium injury and inflammation along with increased NO synthesis by leukocytes.^(43)^ This series of events promotes increased lung vascular permeability and edema, in addition to apoptosis and inflammation. In addition to these events, oxidative stress occurs,^(44)^ which promotes lung injury and causes respiratory insufficiency.^(45)^

In systemic infection, the bacterial antigen reaches the lung level; to combat these microorganisms in the respective tissue, leukocyte infiltration, especially neutrophils, is required.^(46)^ These neutrophils increase myeloperoxidase (MPO) enzyme activity and, through oxidative damage, fight the invading agent.

Additionally, greater neutrophil activity generates an increase in the production of ROS,^(44)^ with an emphasis on the superoxide anion,^(47)^ which causes oxidative lung damage in an experimental model of sepsis.^(44)^

Dysfunction in respiratory capacity is one of the main events responsible for causing systemic dysfunction in an infectious condition, inducing oxygen deprivation secondary to respiratory insufficiency, which is responsible for dysfunction and cell death. This oxygen deprivation can affect cellular glucose metabolism, inducing a higher production of lactate; worsening of the infectious condition is characterized by elevated lactate levels in the blood circulation,^(18)^ with a concomitant decrease in iHSP70 in the lung tissue.^(7)^

Treatment with eHSP70 promotes a decrease in ROS production by neutrophils and monocytes exposed to the bacterial antigen, attenuating the oxidative damage caused by infection.^(44)^ In addition to this protective effect, HSP70 is able to decrease permeability and improve vascular integrity in infected lung tissue, reducing blood leakage and pulmonary edema. This cytoprotective function of HSP70 improves respiratory capacity in sepsis by attenuating cellular dysfunction and decreasing oxidative damage in lung tissue, which leads to less local injury in sepsis.^(45)^

### HSP70, oxidative stress and liver dysfunction in sepsis

The liver plays a key role in sepsis and participates in the defense of the organism, the antiinflammatory/proinflammatory balance, tissue repair and coagulation.^(48)^

Bacterial infection in hepatic tissue causes a decrease in autophagy in this tissue, generating lipid accumulation in hepatocytes, and impairs lipid metabolism homeostasis during sepsis in an experimental model with aged mice.^(49)^ This steatosis causes functional impairment in the tissue, causing an elevation in glutamic oxaloacetic transaminase (GOT) and glutamic pyruvic transaminase (GPT) enzyme levels, which, in turn, is associated with increased liver apoptosis and mortality from sepsis.^(25,34)^

Oxidative stress plays an important role in liver dysfunction in sepsis, with a clinical picture of classic liver dysfunction (elevated GOT and GPT) combined with decreased activity of the antioxidant enzymes SOD and CAT; additionally, an increase in free radicals can be observed, a process that is associated with increased inflammation in this tissue and higher mortality from the disease.^(50)^ Additionally, in sepsis, the liver is affected by oxidative stress, which leads to tissue damage and causes NF-κB activation, inducing increased synthesis, proinflammatory cytokines and cell apoptosis, leading to liver injury and dysfunction.^(33)^

Liver dysfunction in sepsis is related to a decrease in the cytoprotection exerted by iHSP70 in the respective tissue, as the decrease in the hepatic expression of this protein confirms its dysfunction.^(33)^ Considering that the decrease in iHSP70 may occur by cell lysis or active export by cells under extreme stress, the consequent increase in eHSP70 may be related to an increase in plasma concentrations of proinflammatory cytokines^(51)^ because eHSP70 acts as chaperone; i.e., it is recognized by receptors that trigger proinflammatory responses.^(52)^ In turn, treatment with subcutaneous HSP70 immediately after the onset of sepsis in an experimental model produces decreased levels of lesion markers and hepatocyte apoptosis.^(25)^

### HSP70, oxidative stress and urinary/renal dysfunction in sepsis

Systemic infection, on which the pathophysiology of sepsis is based, is capable of causing inflammation, apoptosis and functional impairment of renal tissue.^(53)^ The disease also cause a proinflammatory response involving the immune cells present in the kidneys. As in other tissues, the bacteria present in the genesis of the infection interact with Toll-like receptor (TLR)-4, present in leukocytes, in addition to RAGE, leading to the synthesis of proinflammatory cytokines and cell apoptosis.^(54)^

After the cascade of intracellular events that follow the signaling of immune cells and renal vascular endothelium, tissue inflammation and renal cell apoptosis occur.^(54)^ This local impairment leads to acute kidney injury, which is associated with the sudden reduction in glomerular filtration and evidenced by an increase in serum creatinine levels.^(16)^ In sepsis, there is an inflammatory response and immune dysfunction, which progress from a hyperdynamic state to a hypodynamic phase.^(12)^ In this phase, a reduction in renal blood flow and hypoperfusion results in low oxygen demands that, in a prolonged manner, induce tubular epithelial cell injury, apoptosis and acute tubular necrosis.^(55)^

Oxidative stress is present in the pathophysiology of renal injury in sepsis, and renal injury is related to mortality from the disease in experimental models.^(28)^ This renal injury in sepsis causes an increase in residue from tissue oxidative damage in the urine.^(16)^ Furthermore, renal tissue injury allows exacerbated protein extravasation, such as eHSP70 (uHSP70), along with decreased renal function.^(16,56)^ The presence of this protein in the urine occurs due to the export of inflammatory cells that migrated to the site of infection. Furthermore, the presence of uHSP70 may be caused by the extravasation of damaged epithelial cells in the kidney or urinary tract.^(56)^

Immunotherapy in an experimental treatment for sepsis. In an experimental model, the administration of intravenous glutamine at a dose of 0.75g/kg 1 hour after the onset of sepsis induced by cecal ligation and perforation (CLP) resulted in renal injury attenuation. This treatment increased the expression of renal iHSP70, which was associated with a decrease in the severity of renal injury, as evidenced by lower RAGE and TLR-4 receptor expression, resulting in lower NF-κB activity and cell injury. This series of could result in a lower mortality rate.^(54)^

## CONCLUSION

Oxidative stress and decreased iHSP70 are associated with systemic dysfunction in sepsis. HSP70 exerts cytoprotective activity and is related to functional improvement and can be considered a therapeutic target for the treatment of sepsis.
